# 1885. Resultados clínicos del tratamiento antituberculoso en pacientes oncológicos entre 2011-2022 en Cali Colombia

**DOI:** 10.1093/ofid/ofad500.1713

**Published:** 2023-11-27

**Authors:** Sofía Alexandra Montes-Tello, José Fernando García-Goez

**Affiliations:** Fundación Valle del Lili, Cali, Valle del Cauca, Colombia; Fundación Valle del Lili, Cali, Valle del Cauca, Colombia

## Abstract

**Background:**

Tuberculosis infection represents a clinical challenge despite the fact that treatment can cure 85% of cases because successful outcomes can vary due to other comorbidities such as cancer, during which pharmacological interactions, adverse effects and decreased adherence to treatments are common. This study aims to describe the characteristics and outcomes of patients with active tuberculosis and cancer from a referral hospital.

**Methods:**

A retrospective observational study was conducted, which included patients who were diagnosed with cancer requiring oncological treatment during tuberculosis treatment management, as well as patients who were undergoing oncological management and were diagnosed with active tuberculosis during this process at a referral hospital in southwestern Colombia between 2011-2021.

**Results:**

Fifty-eight patients with tuberculosis and cancer were included. The median age of the patients was 60 years (IQR: 42 – 67), 31% were women and 75% of the cases were pulmonary tuberculosis. Hematological neoplasms accounted for 62% of cases and the intention of oncological therapy was curative in 89% of cases. The most common clinical decision was to administer antituberculous and oncological therapy simultaneously in 46% of cases and the antituberculous regimen was modified in 34% of cases. The success rate of antituberculous treatment was 60% and 28% of cases died.

**Conclusion:**

It is necessary to consider screening for active and latent tuberculosis as a priority in patients with neoplasia diagnoses and ensure prophylactic management.

**Disclosures:**

**All Authors**: No reported disclosures

